# Clozapine Prescribing in General Hospitals: A Second-Cycle Service Evaluation Following Quality Improvement Interventions

**DOI:** 10.1192/bjo.2026.11598

**Published:** 2026-06-30

**Authors:** Dhaveena Rajathevan, Hanna Tee, Namrata Soni, Kara Hosegood

**Affiliations:** 1 Dorset Healthcare University NHS Foundation Trust, Poole, United Kingdom; 2 University Hospitals Dorset, Bournemouth, United Kingdom

## Abstract

**Aims:**

Clozapine is an effective medication for adults with treatment-resistant schizophrenia. However, its safe prescribing and monitoring can be challenging during General Hospital admissions. An initial service evaluation conducted in 2023–2024 identified inaccurate documentation of clozapine in community records, delayed referrals to Liaison Psychiatry and inconsistent monitoring of clozapine-related risk factors. In response, quality improvement interventions were implemented, including local education, a Trust-wide clozapine policy for General Hospitals and a clozapine admission checklist. This second-cycle service evaluation aimed to assess whether these interventions improved the safety and consistency of clozapine prescribing and monitoring.

**Methods:**

A retrospective review was conducted of patients prescribed clozapine in the community who were admitted to two General Hospitals in England between March 2024 and February 2025. There were 66 admissions involving 53 patients. Electronic records from community services, General Hospitals and Mental Health services were reviewed. Data collected included the accuracy of clozapine documentation on community records, whether referrals to Liaison Psychiatry were made and the timeliness of referral and review. Liaison Psychiatry documentation was reviewed to determine whether key clozapine-related factors were considered, including full blood count monitoring, medication concordance, smoking status, bowel function, physical health concerns, medication interactions, signs of clozapine toxicity and advice regarding plasma level monitoring.

**Results:**

Clozapine prescriptions were correctly documented in community records in 26% (17/66) of admissions, compared with 16% in the first cycle. On admission, 79% (52/66) of patients were referred to Liaison Psychiatry, with a reduction in mean time to referral from 41.07 hours to 32 hours. Of those referred, 87% (54/62) were reviewed within 24 hours, compared with 68% in the first cycle.

Within Liaison Psychiatry reviews, documentation rates were highest for medication concordance and physical health concerns (both 88%) and full blood count monitoring (78%). Documentation was lower for medication interactions (59%), bowel movements (50%), signs of clozapine toxicity (33%) and smoking status (19%). Advice regarding plasma level monitoring was documented in 40% of cases.

**Conclusion:**

This second-cycle service evaluation demonstrates improvements in referral rates to Liaison Psychiatry, timeliness of review and the quality of Liaison Psychiatry documentation following targeted quality improvement measures. However, challenges remain in the consistent documentation of clozapine prescriptions in community records and routine assessment of smoking status and signs of toxicity. Further quality improvement initiatives and repeat evaluation cycles are recommended to support safe and consistent clozapine management in General Hospitals.

